# Clinical and Demographic Characteristics of Herpetic Keratitis Patients—Tertiary Centre Experience

**DOI:** 10.3390/medicina60040577

**Published:** 2024-03-31

**Authors:** Petra Grubešić, Igor Jurak, Tea Čaljkušić-Mance, Andrej Belančić, Aron Grubešić

**Affiliations:** 1 Department of Ophthalmology, Clinical Hospital Center Rijeka, Krešmirova 42, 51000 Rijeka, Croatia; tea.mance.caljkusic@uniri.hr; 2 Department of Biotechnology, University of Rijeka, Ul. Radmile Matejčić 2, 51000 Rijeka, Croatia; igor.jurak@biotech.uniri.hr; 3 Department of Clinical Pharmacology, Clinical Hospital Center Rijeka, Krešimirova 42, 51000 Rijeka, Croatia; a.belancic93@gmail.com; 4 Department of Basic and Clinical Pharmacology with Toxicology, Faculty of Medicine, University of Rijeka, Braće Branchetta 20, 51000 Rijeka, Croatia; 5 Department of Internal Medicine, Clinical Hospital Center Rijeka, Krešimirova 42, 51000 Rijeka, Croatia; aron.grubesic@uniri.hr; 6 Department of Internal Medicine, Faculty of Medicine, University of Rijeka, Krešimirova 42, 51000 Rijeka, Croatia

**Keywords:** cornea, epithelial ulceration, herpes simplex keratitis, HSV-1, recurrence

## Abstract

*Background and Objectives*: Herpes simplex keratitis (HSK) is the leading infectious cause of corneal damage and associated loss of visual acuity. Because of its frequent recurrence, it represents a major health problem; thus, timely and accurate diagnosis is the key to successful treatment. To enable this, we aimed to determine HSK patients’ demographic and clinical features. *Materials and Methods*: This prospective study included 55 patients diagnosed with HSK between March 2019 and August 2022 at the Department of Ophthalmology, Clinical Hospital Rijeka. *Results*: We found that HSK is most prevalent in the elderly, with 72.73% of patients older than 60. The most common HSK types were dendritic (HSK-D; 43.64%) and stromal with epithelial ulceration (HSK-SEU 23.64%). HSK recurrences occurred in 65.45% of patients, with most having two to five recurrences (55.56%). Visual acuity at presentation (65.5%) and after treatment (50.9%) was mostly in the 20/50 range. The longest period until the disease symptoms were resolved was in the group with stromal HSK without epithelial ulceration (HSK-SnEU), for which symptoms lasted more than 11 weeks in 87.5% of patients. The overall incidence of HSK-related complications was high (85.45%), with 76.4% of patients having corneal scarring. The average time from symptom to treatment was 15.78 days. Interestingly, we observed a strong seasonality in the incidence of HSK, which was most prevalent in the colder months, with 63.6% of cases occurring between October and March. *Conclusions*: To the best of our knowledge, this is the first prospective study in Croatia, and one of the few in Europe, to describe the demographic and clinical features of HSK patients. We found that HSK is most common in the elderly population, with its dendritic form as a clinical presentation. We have shown that HSK is prone to recurrence and secondary complications, with a worryingly long time between symptom and treatment, indicating the need for diagnostic testing in routine practice.

## 1. Introduction

Herpes simplex virus 1 (HSV-1) is a member of the *Ortoherpesviridae* family, which includes a number of important human pathogens, including HSV-1, herpes simplex virus 2 (HSV-2), and varicella-zoster virus (VZV). The incidence of herpetic eye disease is high, and it has been estimated that more than 1.8 million cases occur annually worldwide [1]. It has also been shown that in 2016 alone, approximately 230,000 people had a newly acquired unilocular visual impairment associated with HSK [1]. In addition, it is essential to note that HSK represents a significant economic burden, equating to around USD 17.7 billion in expenditure related to treating the disease and its complications [2].

The main characteristic of HSV-1 is that it establishes a latency period in the sensory and autonomic ganglia after a productive infection. Under various stimuli, such as stress, the virus can reactivate from latency and cause recurrent disease. Reactivations from latency and recurrences are the leading cause of disease and morbidity caused by these viruses [3,4]. However, treatment options are only available for productive infections [3,4,5].

Primary ocular HSV infection is usually asymptomatic but may manifest as conjunctivitis, blepharitis, lip lesions, and other manifestations in 1–6% of patients, depending on the immunologic status of the host [5]. Concomitantly, under the immunocompromised conditions of the host, there is recurrent viral replication, causing target infection by viral migration along the neural axis [5,6]. It has been estimated that 40% of patients harbouring latent infection have had at least one recurrence within a 5-year period [6,7].

HSV infection can affect all corneal layers and divide them into different groups. The epithelial form (HSK-E) is the most common and is caused by active viral replication in the superficial corneal layer. Depending on how fluoresceine staining is viewed with a biomicroscope, HSK-E is divided into dendritic (HSK-D) or geographical form (HSK-G).

The stromal form of HSK (HSK-S) clinically presents as a whiteish stromal blurring of the cornea with or without fluorescein-coloured epithelial damage and, in some cases, with characteristic Descemet folds [5]. It is subdivided according to the presence of epithelial ulcerations (HSK-SEU when present and HSK-SnEU when absent). The endothelial form (HSK-En) of HSK presents clinically with stromal oedema and endothelial dysfunction, as well as keratic precipitates in the absence of significant anterior uveitis. It is often associated with increased intraocular pressure due to inflammation at the level of the trabecular network [8,9].

It is well known that HSK, especially in the later stages, can mimic other causes of keratitis, which is why it is often referred to as “chameleon disease” in the literature [10]. The modality of HSK treatment depends on the affected corneal layer, but the essence of any therapy is an adequate antiviral dose, in most cases, combined with corticosteroids. Treatment of the epithelial HSK form includes a therapeutic dose of an antiviral agent for seven to ten days for the dendritic form, whereas, for the geographical form, treatment lasts for fourteen to twenty-one days. On the other hand, HSK-SEU is treated with a limiting dose of topical corticosteroids and a therapeutic dose of antiviral agents for seven to ten days. Depending on the clinical course, this can be further consolidated by a prophylactic dose of the same agents. Furthermore, HSK-SnEU is treated with a higher therapeutic dose of topical steroids and a prophylactic dose of antivirals, with further tapering of steroid dose of up to ten weeks [5]. Lastly, HSK-En is treated with a therapeutic dose of topical steroids and a therapeutic dose of antivirals for seven to ten days with a later prophylactic dose.

The length of the treatment course itself depends on the disease course, with there being no referent clinical studies available to provide optimal treatment length information. It is important to note that however long topical steroid treatment lasts, it should be accompanied by adequate antiviral prophylaxis [5].

In most parts of Europe, molecular HSK diagnosis is performed rarely and sporadically and is yet to be introduced into everyday protocols. HSK diagnosis is most commonly established based on clinical experience and presentation, which is inadequate due to the kaleidoscope of different presentations, as shown above. Even though the first HSK studies are 30 years old, HSK is still an important research subject, as there is insufficient information on its incidence, prevalence, clinical course, and antiviral resistance for ascertaining morbidity and developing disease control strategies correctly [11].

This study aimed to further investigate the clinical and demographic characteristics of patients with HSK to establish clinical procedures that would allow for the accurate diagnosis and timely treatment of the disease.

## 2. Materials and Methods

This prospective study included 55 patients diagnosed with HSK between March 2019 and August 2022. We included patients treated in the Ophthalmology department at the Clinical Hospital of Rijeka diagnosed with HSK based on established clinical criteria. HSK was classified according to the American Academy of Ophthalmology, which divides HSK into five subtypes [5]. The criterion for the dendritic form of epithelial HSK is a linear epithelial lesion with terminal bulbi. In contrast, the geographical form is characterised by a loss of the linear form and further ulcer enlargement, thus assuming a typical geographical phenotype.

The epithelial HSK outline and borders are properly visualised following fluorescein colouring of the cornea. The clinical features of stromal HSK result from a stromal infiltrate caused by intrastromal reaction and possible oedema. The stromal HSK form is further classified based on whether epithelial ulceration is present, visualised via fluorescein colouring. Notably, stromal HSK with an epithelial defect is indicative of a greater risk of corneal melting and perforation. Clinical signs of HSK endothelial form are corneal precipitates with concurrent stromal oedema caused by endothelial decompensation without the presence of stromal infiltrates or neovascularisation typically found in stromal HSK.

Visual acuity was measured via a Snellen chart on a 6-metre range (20 feet) at patient presentation and after treatment ended. The Snellen chart is a frequently employed eye chart for assessing visual acuity. The numerator indicates the distance, measured in feet, at which you position yourself from the chart. The denominator is the reading distance for an individual with typical vision who can read the same line as you did accurately. An individual with 20/20 vision can perceive the same visual acuity as an average person while viewing an eye chart from a distance of 20 feet. In comparison, 20/40 vision means that at 20 feet away, you can see letters that would usually be seen at 40 feet.

Patients were divided into two groups depending on the level of visual acuity. The first group included patients ranging from 20/20 to 20/40, and the second group included patients ranging from 20/50 to 20/200.

The clinical parameters we investigated were age, sex, frequency of recurrence, HSK type, days from presentation, treatment lengths, visual acuity at presentation and at the end of treatment, and the complications present following the resolution of HSK.

In our research, HSK diagnostics were based on clinical findings or a previous anterior segment infection history classified as HSK. The exclusion criteria were anterior segment infections likely caused by bacteria or fungi.

### 2.1. Summary of Treatment Protocols According to HSK Type

All 55 patients were treated depending on HSK type based on the latest American Academy of Ophthalmology guidelines, with established recommendations for the duration of HSK treatment [5].

Treatment was conducted depending on HSK type as follows:

- HSK-D—acyclovir: 400 mg 3–5 times daily for 7–10 days or acyclovir ophthalmic gel instillation of 1 drop into the affected eye(s) 5 times daily for seven days.
- HSK-G—acyclovir: 800 mg 5 times daily for 14–21 days or acyclovir ophthalmic gel instillation of 1 drop into the affected eye(s) 5 times daily for seven days.
- HSK-SnEU—Topical corticosteroid six times daily tapered over more than ten weeks with acyclovir: 400 mg twice daily prophylaxis until corticosteroid is tapered out.
- HSK-SEU—Topical corticosteroid two times daily tapered over more than ten weeks with acyclovir 800 mg 3–5 times daily for 7–10 days followed by acyclovir 400 mg twice daily prophylaxis until corticosteroid is tapered out.
- HSK-En—Topical corticosteroid six times daily tapered over greater than 10 weeks with acyclovir: 400 mg 3–5 times daily for 7–10 days followed by acyclovir 400 mg twice daily prophylaxis until corticosteroid is tapered out.

Nevertheless, the duration of treatment is flexible and is dependent on the clinical features and disease course. Furthermore, due to this study’s high number of recurrent cases, further prophylactic antivirals were continued for up to six months following initial treatment. In our study, we divided the length of treatment into periods ranging from one, two, or three weeks; one, two, or three months; and four or more months.

### 2.2. Statistical Analysis

Statistical analysis was performed using Microsoft Excel (Microsoft Office—version 16.78.3) and MedCalc v14.8.1 (MedCalc Software bvba, Ostend, Belgium). Absolute and relative frequencies, measures of central tendency alongside measures of spread, were used to present the data. The Kolmogorov–Smirnov test was used to assess the normality of distribution. The Wilcoxon test was used to determine the difference in visual acuity before and after treatment. The Kruskal–Wallis test was used to compare the differences between the HSK types over time until clinical presentation, treatment duration, and time until resolution. Furthermore, to compare the differences in complication frequencies per HSK group, a χ^2^-test was used. All statistical tests were two-tailed and had a 95% CI. Overall, the criterion for statistical significance was set at *p* < 0.05.

### 2.3. Ethical Approval and Good Clinical Practice

This research was conducted according to all applicable guidelines, aiming to ensure the proper conducting of investigations and patient safety while considering good clinical practice. All patients signed informed consent for their participation and for the further publishing of their data. The Clinical Medical Center Rijeka Ethics Committee, Croatia, reviewed and approved all procedures and objectives.

## 3. Results

### 3.1. Patients and Clinical Manifestations of HSK

**Figure 1:**
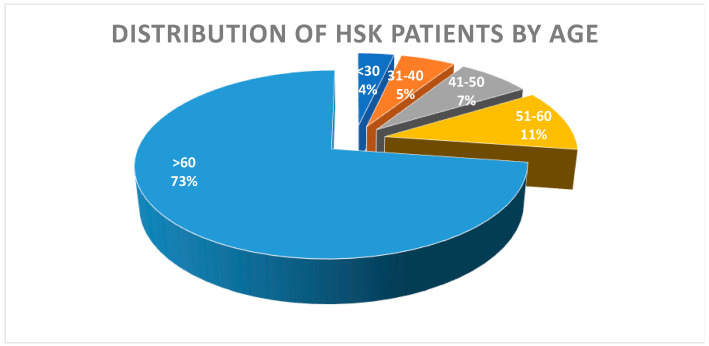
Distribution of patients with diagnosed herpes simplex keratitis (HSK) by age. The diagram presents 55 patients (29 male and 26 female) admitted at the Clinical Hospital Rijeka between March 2019 and August 2022 diagnosed with HSK. The percentage of patients in different age groups (>60—age over 60, etc.) is indicated by different colours, the size of the chart, and the indicated numbers (%).

To characterise HSK’s clinical and demographic characteristics, we analysed a cohort of 55 patients diagnosed with HSK based on clinical presentation and history of HSK. The study included 29 male (52.73%) and 26 female (47.27%) patients. We found no statistical difference in the incidence of HSK between the genders (*p* = 0.537, x^2^ = 0.318). The average age of all patients was 67.13 years, median of 69 (age 20–89), with a mean age of 65.31 for women and 68.76 for men. Our results also show no statistical difference in the presentation of HSK between the genders with respect to age (*p* = 0.569, x^2^ = 0.325). Overall, most patients (40) were older than 60 years (72.73%). There were six patients in the 51–60 years group (10.91%), four patients were in the 41–50 years group (7.28%), three patients were in the 31–40-year group (5.45%), and two patients were younger than 30 years (3.64%) (Figure 1).

Of the 29 male patients, 14 were diagnosed with HSK-D (48.27% of males, 43.6% overall), 2 with HSK-G (6.89%, 7.3% overall), 7 with HSK-SEU (24.13%, 23.64% overall), 3 with HSK-SnEU (10.34%, 14.6% overall), and 3 with HSK-En (10.34%, 10.9% overall). On the other hand, of the 26 female patients, 10 were diagnosed with HSK-D (38.46%), 2 with HSK-G (7.69%), 6 with HSK-SEU (23.07%), 5 with HSK-SnEU (19.23%), and 3 with HSK-En (11.53%). Interestingly, although HSK-En can be associated with anterior uveitis, we did not detect it in any of our patients.

The distribution of patients by age and HSK type is shown in Table 1.

Interestingly, in the follow-up period, 36 out of 55 patients (65.5%) had a recurrence of HSK, while 19 patients (34.6%) presented with HSK as the first manifestation. The frequency of recurrent disease varied significantly among the patients. The patients commonly had two to five recurrent diseases (i.e., 56% N = 20/55). A single recurrence occurred in 25% (N = 9/55) of patients, while 5–10 and more than 10 recurrences occurred in 11% (N = 4/55) and 8% of patients (N = 3/55), respectively.

### 3.2. Visual Acuity of Patients with HSK Improves after Treatment with Antivirals

**Figure 2:**
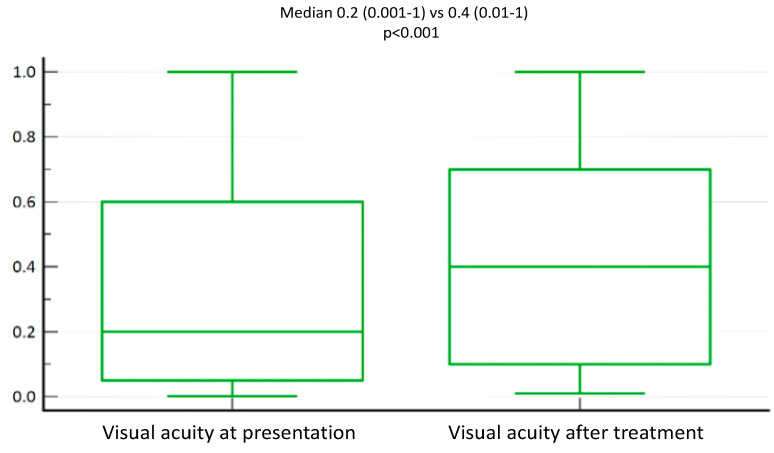
Visual acuity in HSK patients improves significantly after treatment. Based on the latest guidelines from the American Academy of Ophthalmology, visual acuity was measured at presentation and following treatment. Number of patients = 54. Visual acuity at presentation (left bar) and after treatment (right bar). The median values of measured visual acuity in both groups are indicated (median 0.2 (0.001–1) and 0.4 (0.01–1), respectively; *p* < 0.001). Note: Statistical analysis was performed with the Wilcoxon test. Visual acuity was measured via the Snellen table chart.

To analyse the extent of visual impairment during the onset of the disease, we first measured visual activity in all patients. At the onset of the disease, visual acuity was reduced in most patients, i.e., it was 20/50 (N = 36/54, 65.5%), while 33% of patients had visual acuity between 20/20 and 20/40 (N = 18/54, 32.7%). One patient was blind and was excluded from the analysis. However, visual acuity after treatment was notably satisfactory and in the 20/20 to 20/40 range in 26 patients, while most patients (N = 28, 50.9%) presented with 20/50. We found that there was a statistically significant improvement in visual acuity after treatment (Median 0.2 (0.001–1) vs. 0.4 (0.01–1), *p* < 0.001) (Figure 2).

Next, we analysed visual acuity by different clinical manifestations of HSK. Briefly, visual acuity at presentation was in the 20/20–20/40 (1–0.5) range for 14 patients (58.3%) with HSK-D, 1 patient with HSK-G (25%), and 3 patients with HSK-SEU (23.1%). All patients with HSK-SnEU and those with HSK-En were in the poorer visual acuity group at presentation, ranging from 20/50 to 20/200 (0.4–0.1). Furthermore, ten patients (76.9%) from the HSK-SEU group, as well as three patients (75%) with HSK-G and ten patients (41.7%) with HSK-D, were also in the poorer visual acuity group.

Visual acuity after treatment, based on the latest Guidelines from the American Academy of Ophthalmology, Ref. [5], was in the 20/20–20/40 range for 18 patients (75%) with HSK-D. With most other patients, we found poorer visual acuity ranging from 20/50 to 20/200, specifically in three patients with HSK-G, ten with HSK-SEU, five with HSK-SnEU, and five with HSK-En.

### 3.3. Duration of Treatment and Time until HSK Resolution

**Figure 3:**
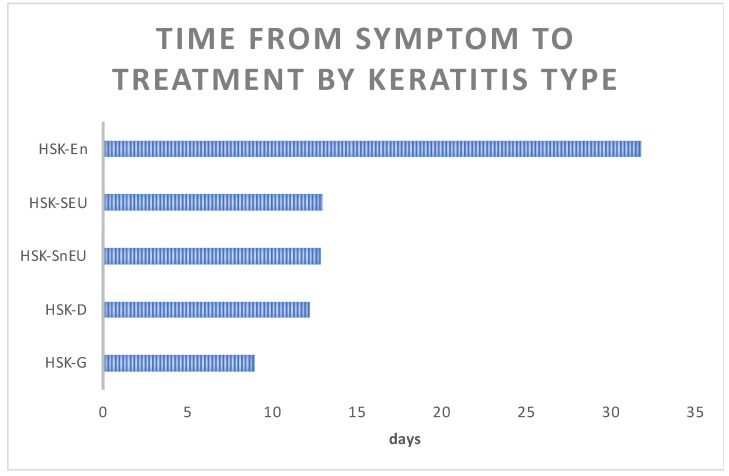
Time from symptom to treatment by keratitis type. Average time from symptom onset to HSK diagnosis and commencement of treatment between various HSK types (HSK-En—endothelial HSK, HSK-SEU—stromal HSK with epithelial ulceration, HSK-SnEU—stromal HSK without epithelial ulceration, HSK-D—dendritic HSK, HSK-G—geographic HSK).

We aimed to determine whether response to treatment was associated with delays in treatment due to the time required for accurate diagnosis. The average time from symptom onset to HSK diagnosis and treatment initiation was 15.78 days, similar to most HSK types. The shortest time to diagnosis, under 10 days, was found in the HSK-G group, and the shortest time was about 12 days for most of the other HSK types. However, HKS-En drastically differed from the other forms, requiring up to 35 days (Figure 3).

**Figure 4:**
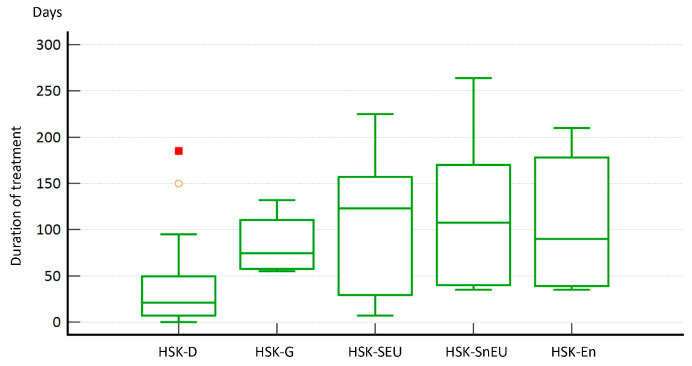
Length of treatment based on the latest guidelines from the American Academy of Ophthalmology. HSK-D required a significantly shorter treatment duration (*p* = 0.006) compared to the other HSK types. Notes: Statistical analysis was performed with the Kruskal–Wallis test. Red marks represent outliers.

On the other hand, it is not surprising that the duration of treatment also varied considerably between the different forms of HSK. For most patients (N = 30, 54.6%), treatment with acyclovir (w or w/o corticosteroids) lasted longer than six weeks. Briefly, in 58.3% of dendritic HSK patients, we concluded treatment within 4–6 weeks, with the longest treatment being (period to the resolution of symptoms) in the HSK-SnEU group (87.5%), lasting for more than 11 weeks. Furthermore, several other patient groups had treatment lasting for more than 11 weeks, such as the HSK-SEU (77%), HSK-G form (75%), and HSK-En (66.6%) groups (Figure 4). These results suggest that early diagnosis does not necessarily correlate with the duration of treatment (e.g., HSK-G was diagnosed quickly, but treatment took a long time) but rather depends on the type of HSK. Nevertheless, accurate diagnosis is crucial for appropriate treatment.

**Figure 5:**
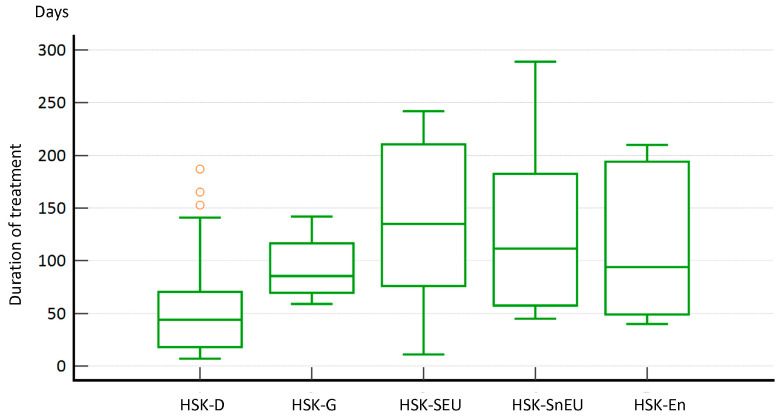
Days to symptom resolution between different HSK types. Notes: Statistical analysis was performed with the Kruskal–Wallis test. Red marks represent outliers.

As expected and similar to the observed difference in treatment duration between HSK-D and the other HSK types (*p* = 0.006), we observed a statistically significant difference (*p* = 0.018) between HSK-D compared to all other HSK types in terms of days required for symptom resolution (Figure 5).

### 3.4. Frequency of Complications

The healing of HSK is often associated with additional clinical complications and morbidities due to triggered immunity and various cellular signalling pathways, including the stimulation of neovascularisation. We were interested in investigating the incidence of corneal scaring, neovascularisation, persistent epithelial defects, and glaucoma in different HSK types after HSK healing. In summary, we found that 11 out of 21 patients (52.4%) with HSK-S had neovascularisation compared to 2 out of 34 (5.9%) in all other groups, which is statistically significant (χ^2^ =15.261, *p* < 0.001). Furthermore, 7 out of 28 patients (25%) in the HSK-E group had persisting epithelial defects compared to 1 out of 27 (3.7%) in the remaining groups (χ^2^ =4.926, *p* = 0.026). In addition, 3 out of 6 patients (50%) with HSK-En had glaucoma, as opposed to 2 out of 49 (4.1%) in the remaining groups, which is also statistically significant (χ^2^ =13.357, *p* < 0.001) (Table 2). The overall incidence of HSK-related complications was high (85.45%), with 76.4% of patients having corneal scarring as the most common complication.

### 3.5. Seasonal Distribution of HSK

**Figure 6:**
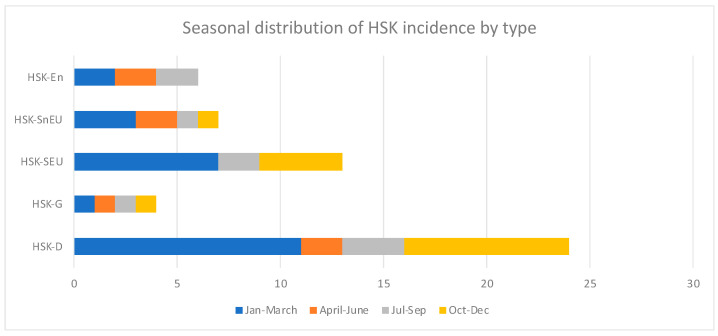
Seasonal distribution of HSK by type.

Numerous individual and environmental factors, such as seasonality, can affect the development of HSK and its recurrence. The distribution of HSK shows two yearly peaks, one in the January to March period (N = 21, 38.2%) and the other in the October to December period (N = 14, 25.5%). The seasonal distribution of HSK incidence by HSK type is depicted in Figure 6.

## 4. Discussion

Despite years of intensive research and the development of effective antiviral agents, herpetic keratitis remains a major health problem. Each year, approximately 1.8 million people worldwide contract HSV keratitis, including more than 40,000 new cases that will lead to severe visual impairment [12]. In addition, it should be noted that HSK represents a significant economic burden, amounting to approximately USD 17.7 billion in expenditure for the treatment of the disease and its complications [2]. Timely and correct diagnosis is the cornerstone for effective treatment and preventing severe morbidity. However, the clinical features of HSK are not well characterised, and diagnosis can be difficult because of its similarity to other infectious diseases [1]. In this study, we aimed to contribute to the characterisation of HSK by carefully analysing a cohort of patients admitted to the Department of Ophthalmology, Clinical Hospital Rijeka, over three years (March 2019–August 2022). We enrolled 55 patients with diagnosed HSK, including 29 males (52.73%) and 26 women (47.27%). Our results suggest there is no apparent difference in the incidence of HSKs between men and women based solely on the number of patients diagnosed. A similar observation was made by Gyu-Nam et al. and Yousuf et al. in their studies, in which the incidence was slightly on the side of the male population [13,14]. However, other studies have found a higher incidence of HSK in the female population, including for primary and recurrent infections [11]. However, because of several limitations in our research, including the need for a national disease registry and integrating public and private hospitals into a standard system, it is impossible to estimate the exact prevalence of HSK in the Croatian population. There is a large discrepancy between different studies in estimating the annual incidence of HSK, which varies between 10 and 300 cases per 100,000 person years [1].

The average patient age was 67.13 years, with more than 72% of patients being older than 60. Only a few patients were younger than 30 (Table 1). This differs somewhat from previous studies, which indicated that most cases occur in adults under 60, while patients over 60 account for only about 25% of cases [15,16]. The older study population is likely due to our Department of Ophthalmology being a tertiary Clinical hospital where patients with recurrent and more difficult clinical conditions are examined and admitted. In contrast, younger and more immunocompetent patients with milder infections are treated in secondary or primary care facilities. Indeed, other studies conducted at the tertiary level, such as the study by Alfaro et al., found the average age of HSK patients to be 62 years [10].

Next, we analysed the types of HSK. In our study, HSK-D was the most common (43.6%), followed by HSK-SEU (23.64%). This observation agrees well with previous studies by others [17]. However, it is essential to note that in some studies, the stromal type is the most common form of HSK, which is explained by changes in the course of the disease, the pathogenesis of which is mediated more by the immune response over time. In other words, the status of the immunologically privileged site is compromised as the disease progresses [18,19]. Peculiarly, in some cases, it is very challenging to precisely subtype the HSK form, particularly in overlapping HSK, and Alfaro et al. reported a combination of epithelial and endothelial forms of HSK to be the most prevalent form of HSK [10].

One of the main features of HSK is its recurrence, and we found that about 65% of the cases admitted were actually recurrent HSK. Moreover, over 55% of patients experienced two to five recurrences during our study (3 years). In the literature, the number of recurrences noted in different studies varies widely, ranging from 20% to 65%. It is difficult to explain such discrepancies, and it might depend on the age of the patients in the study, i.e., strictly focusing on younger patients might yield a different frequency of recurrences than in the older population. Nevertheless, we and others have observed that the intervals between recurrences are shorter when the frequency of recurrences is higher [11,13,14,15]. We did not find any gender-specific differences in the frequency of recurrences, which is in accordance with other studies [13].

One of the essential pieces of information we wanted to obtain from our study concerned treatment, including the correlation between the type of HSK and the duration of treatment and improvement in disease symptoms. Overall, the duration of treatment in our study was more than 11 weeks for most patients (54%), with an average duration of 11.6 ± 9.7 weeks. However, we found that the dendritic form of HSK required the shortest treatment (median 18.73 days, *p* = 0.006) compared to other forms. In addition, we observed a statistically significant difference (*p* = 0.02) in days to resolution between the different forms of HSK, i.e., the dendritic group required fewer days (median = 19.60 days) than the stromal HSK group (median = 35.44 days). In the stromal form of HSK, the main initiators of the inflammatory reaction are T lymphocytes that recognise viral antigens but also cross-react with the autoantigens of the cornea itself [20]. Therefore, in the case of HSK-S, the primary substrate of the disease is a strong inflammatory and autoimmune response rather than viral pathogenesis, making treatment much more challenging and longer [21]. The endothelial form of HSK is also known to be immunologically mediated. It is the form of HSK with the most prolonged time lag between symptoms and correct diagnosis because the clinical presentation is nonspecific [5]. Other reports indicated the duration of treatment from only a week to over eight weeks [6,14]. Such a large discrepancy between different studies is not surprising as there are no universally accepted protocols for the treatment and diagnosis of HSK, and various socioeconomic factors can significantly impact both.

HSK’s pathology is often associated with additional complications, such as neovascularisation, glaucoma, and persistent epithelial defects. It also indicates a chronic disease course and a robust immunologic component of the disease. In our study, more than 85% of patients suffered some additional complications, similar to other reports [13]. In particular, neovascularisation was present in 52.4% of patients with stromal forms of HSK compared to the other groups (*p* < 0.001). Since both forms are associated with stromal clouding, neovascularisation is one of the differential diagnostic indicators of a recurrent stromal form, rather than an endothelial form, of HSK.

Furthermore, persistent epithelial defects were more frequently associated with the HSK-E group when compared to other forms (*p* = 0.026). On the other hand, we observed the highest incidence of glaucoma in those with endothelial forms (i.e., more than 50%) of HSK (*p* < 0.001), indicating that inflammation at the level of the trabecular network is one of the essential clinical features of this form of HSK [2,5].

Timely and correct diagnosis is the cornerstone for successful treatment and recovery, but accurately diagnosing HSK is a significant challenge. In our study, the average time from first symptoms to diagnosis and the application of appropriate therapy was approximately 15 days (15.78 days). The shortest time to diagnosis was found in those with the epithelial form of HSK, probably due to its specific and easily recognisable appearance after staining with a fluorescein test. It is 12 days for the HSK-D form and 9 days for the HSK g form. The stromal form of HSK takes about 13 days, and the diagnosis is often based on anamnestic information about previous HSV eye infections. Diagnosis took the longest for the endothelial form, averaging 32 days. The endothelial form of HSK is nonspecific in its expression, and its appearance may resemble infection with other pathogens, such as VZV or CMV, or autoimmune diseases [5]. Overall, it is clear that HSK cannot be diagnosed simply based on clinical appearance and that this cannot be the only diagnostic tool which we use to make decisions about the treatment. Also, prescribing ineffective therapy, such as various combinations of antibiotic drops and ointments or introducing corticosteroids without first starting with antivirals, can lead to the masking of the clinical picture, as well as damage to the surface of the conjunctiva and cornea due to toxicity. The delayed initiation of proper therapy may lead to irreversible changes in corneal structure, decrease corneal transparency, and result in scar formation, ultimately leading to poorer visual acuity [5,10].

Finally, our study observed a higher incidence of HSK during the cold season. However, it is difficult to establish a specific pattern because of the relatively small number of patients. Similarly, other studies have indicated seasonality [14,15]. Indeed, a study conducted in Tokyo, Japan, showed a correlation between environmental factors and HSK and its recurrence, indicating that cold temperatures are one of the potential triggers for the reactivation of HSV-1 from latency [22].

For example, Yousuf et al., in their study, found that 46% of HSK cases arose between January and March, whereas Kabra et al. found that 59% of cases appeared between June and December [14,15].

Nonetheless, numerous individual and environmental factors can affect the development of HSK and its recurrence, and these factors should be carefully analysed in future studies.

Our study has limitations; for instance, we should have examined corneal sensitivity, as this could have provided helpful information. Furthermore, we will require a larger patient cohort to confirm and expand the results of our study. Also, misdiagnosis in some patients could have occurred due to the diagnoses being made based on clinical presentation and patients’ history of previous HSK recurrences.

## 5. Conclusions

HSV-1 is the most common infectious cause of corneal blindness, and the correct diagnosis and treatment of HSK remains a challenge. In this study, we included 55 patients diagnosed with HSK at the Department of Ophthalmology of the Clinical Hospital Rijeka, the main tertiary care institution in Promorska–Goranska County. As expected, HSK was most prevalent in the elderly population (72% of patients were older than 60). The most common form of HSK was dendritic, followed by the stromal form with epithelial ulceration (HSK-SnEU). Most patients suffer from recurrent disease and have a long-term visual impairment. Significantly, patients’ visual acuity improved after treatment, but in some forms of HSK symptoms, treatment lasted longer than 11 weeks. In addition, we observed a robust seasonal dependence on the occurrence of HSK. Our study is the first of its kind in Croatia, and one of the few in Europe in the last several decades, to report the demographic and clinical features of HSK patients. As such, it is an important step towards standardising procedures in the tertiary hospital sector and beyond. Furthermore, the results of our study are largely consistent with those of previously published studies, suggesting that (a) there are no specific demographic characteristics that distinguish our studied population and that (b), more importantly, standard procedures have been established that can serve as a template for larger population studies.

## Figures and Tables

**Table 1:** Distribution of patients by age and HSK type.

Keratitis Type	< 30 Years	31–40 Years	41–50 Years	51–60 Years	> 60 Years	N	%
HSK-D	1 (4.2%)	3 (12.5%)	1 (4.2%)	4 (16.7%)	15 (62.5%)	24	43.6
HSK-G	0	0	0	0	4 (100%)	4	7.3
HSK-SEU	0	0	0	3 (23%)	10 (74%)	13	23.6
HSK-SnEU	0	0	1 (12.5%)	1 (12.5%)	6 (75%)	8	14.6
HSK-En	0	1 (16.7%)	1 (16.7%)	0	4 (66.7%)	6	10.9
Total	1 (4.2%)	4 (7.3%)	3 (5.5%)	8 (14.54%)	39 (71%)	55	100

Abbreviations: HSK—herpes simplex keratitis, HSK-En—endothelial HSK, HSK-SEU—stromal HSK with epithelial ulceration, HSK-SnEU—stromal HSK without epithelial ulceration, HSK-D—dendritic HSK, HSK-G—geographic HSK.

**Table 2:** The frequency of complications is based on the type of HSK.

Complication Type	HSK-D (N = 24)	HSK-G (N = 4)	HSK-SEU (N = 13)	HSK-SnEU (N = 8)	HSK-En (N = 6)	Total (N = 55)
No complications	8 (33.3%)	0 (0%)	0 (0%)	0 (0%)	0 (0%)	8 (14.6%)
Corneal scarring	12 (50.0%)	4 (100%)	12 (92.3%)	8 (100%)	6 (100%)	42 (76.4%)
Glaucoma	1 (4.2%)	0 (0%)	0 (0%)	1 (12.5%)	3 (50%)	5 (9.1%)
Cataract	0 (0%)	2 (50%)	4 (30.8%)	3 (37.5%)	3 (50%)	12 (21.8%)
Persisting epithelial defect	6 (25%)	1 (25%)	0 (0%)	1 (12.5%)	0 (0%)	8 (14.6%)
Corneal neovascularisation	1 (4.2%)	1 (25%)	8 (61.5%)	3 (37.5%)	0 (0%)	13 (23.6%)

Abbreviations: HSK—herpes simplex keratitis, HSK-En—endothelial HSK, HSK-SEU—stromal HSK with epithelial ulceration, HSK-SnEU—stromal HSK without epithelial ulceration, HSK-D—dendritic HSK, HSK-G—geographic HSK.

## Data Availability

Data are contained within the article.

## References

[1] McCormick I., James C., Welton N.J., Mayaud P., Turner K.M.E., Gottlieb S.L., Foster A., Looker K.J. (2022). Incidence of Herpes Simplex Virus Keratitis and Other Ocular Disease: Global Review and Estimates. Ophthalmic Epidemiol..

[2] Labib B.A., Chigbu D.I. (2022). Clinical Management of Herpes Simplex Virus Keratitis. Diagnostics.

[3] Roizman B., Knipe D.M., Whitley R.J., Fields B.N. (2013). Herpes Simplex Viruses. Fields Virology.

[4] Roizman B., Whitley R.J. (2013). An Inquiry into the Molecular Basis of HSV Latency and Reactivation. Annu. Rev. Microbiol..

[5] Lee White M., Chodosh J. (2014). Herpes Simplex Virus Keratitis: A Treatment Guideline—2014.

[6] Erdem E., Harbiyeli I.I., Ozturk G., Oruz O., Acikalin A., Yagmur M., Ersoz R., Yarkin F. (2020). Atypical herpes simplex keratitis: Frequency, clinical presentations and treatment results. Int. Ophthalmol..

[7] Wilhelmus K.R., Dawson C.R., Barron B.A., Bacchetti P., Gee L., Jones D.B., Kaufman H.E., Sugar J., Hyndiuk R.A., Laibson P.R. (1996). Risk factors for herpes simplex virus epithelial keratitis recurring during treatment of stromal keratitis or iridocyclitis. Herpetic Eye Disease Study Group. Br. J. Ophthalmol..

[8] Claoue C.M., Menage M.J., Easty D.L. (1988). Severe herpetic keratitis. I: Prevalence of visual impairment in a clinic population. Br. J. Ophthalmol..

[9] Chaloulis S.K., Mousteris G., Tsaousis K.T. (2022). Incidence and Risk Factors of Bilateral Herpetic Keratitis: 2022 Update. Trop. Med. Infect. Dis..

[10] Alfaro Rangel R., Lepper S., Szentmary N., Langenbucher A., Seitz B. (2021). Herpes Simplex Virus Keratitis in a University Tertiary Referral Centre—Clinical Features and Surgical Approaches. Klin. Monbl. Augenheilkd..

[11] Miserocchi E., Fogliato G., Bianchi I., Bandello F., Modorati G. (2014). Clinical features of ocular herpetic infection in an italian referral center. Cornea.

[12] Farooq A.V., Shukla D. (2012). Herpes simplex epithelial and stromal keratitis: An epidemiologic update. Surv. Ophthalmol..

[13] Kim G.N., Yoo W.S., Park M.H., Chung J.K., Han Y.S., Chung I.Y., Seo S.W., Yoo J.M., Kim S.J. (2018). Clinical Features of Herpes Simplex Keratitis in a Korean Tertiary Referral Center: Efficacy of Oral Antiviral and Ascorbic Acid on Recurrence. Korean J. Ophthalmol..

[14] Yousuf M., Akhter M. (2018). Sajad. Clinical Spectrum of Herpes Simplex Keratitis in Patients Attending various Health Institutions in North India. J. Med. Sci. Clin. Res..

[15] Kabra A., Lalitha P., Mahadevan K., Prajna N.V., Srinivasan M. (2006). Herpes simplex keratitis and visual impairment: A case series. Indian J. Ophthalmol..

[16] Chaudhary M. (2017). Clinical and Epidemiological Profile of Herpetic Eye Disease in a Tertiary eye Care Center. J. Inst. Med..

[17] Duan R., de Vries R.D., Osterhaus A.D., Remeijer L., Verjans G.M. (2008). Acyclovir-resistant corneal HSV-1 isolates from patients with herpetic keratitis. J. Infect. Dis..

[18] Guo L.L., Zhang Y., Li N., Wang Z.Q., Tian L., Deng S.J., Sun X.G. (2022). Clinical manifestations of 1 015 cases of herpes simplex virus keratitis. Zhonghua Yan Ke Za Zhi.

[19] Shah A., Joshi P., Bhusal B., Subedi P. (2019). Clinical Pattern and Visual Impairment Associated with Herpes Simplex Keratitis. Clin. Ophthalmol..

[20] Wang L., Wang R., Xu C., Zhou H. (2020). Pathogenesis of Herpes Stromal Keratitis: Immune Inflammatory Response Mediated by Inflammatory Regulators. Front. Immunol..

[21] McDonald E.M., Patel D.V., McGhee C.N. (2015). A prospective study of the clinical characteristics of patients with herpes simplex and varicella zoster keratitis, presenting to a New Zealand emergency eye clinic. Cornea.

[22] Araki H., Takamura E., Shinozaki K., Hori S. (2004). Influence of seasonal variation and recurrence of herpes simplex virus keratitis. Investig. Ophth. Vis. Sci..

